# Intraoperative Nerve Action Potential Amplitude and Functional Recovery After Selective Ulnar-to-Musculocutaneous Nerve Transfer (Oberlin Technique)

**DOI:** 10.3390/jcm15072521

**Published:** 2026-03-26

**Authors:** Diana M. Ortega-Hernández, Aroa Casado-Rodríguez, Isabel Fernández-Conejero, Guillermo J. Tarnawski-Español, Julia Miró-Lladó, Joaquin Casañas-Sintes, Manuel Llusá-Pérez

**Affiliations:** 1 Hand and Microsurgery Unit, Traumaunit, Teknon Medical Center, 08022 Barcelona, Spain; dr.casanas@traumaunit.es; 2 Medicine and Translational Research, Faculty of Medicine and Health Sciences, Universitat de Barcelona, 08036 Barcelona, Spain; 3 Human Anatomy and Embryology Unit, Department of Surgery and Medical-Surgical Specialties, Faculty of Medicine (Campus Clínic), Universitat de Barcelona, 08036 Barcelona, Spain; aroa.casado@ub.edu (A.C.-R.); mllusa@ub.edu (M.L.-P.); 4 Department of Intraoperative Neurophysiology, Bellvitge Universitary Hospital, Universitat de Barcelona, 08907 Barcelona, Spain; isabelfc74@yahoo.es (I.F.-C.); juliamirollado@gmail.com (J.M.-L.); 5 Orthopaedic Surgery and Traumatology Department, Hospital Fraternidad-Muprespa Habana, 28036 Madrid, Spain; gjtarnawski@gmail.com

**Keywords:** brachial plexus, nerve transfer, nerve action potential, electromyography, intraoperative neurophysiologic monitoring, Oberlin

## Abstract

**Background:** Predicting functional recovery after selective nerve transfer remains challenging. Intraoperative nerve action potential (NAP) recording is widely used to confirm axonal continuity in peripheral nerve surgery; however, its quantitative prognostic value in selective nerve transfer has not been clearly established. This study evaluated whether intraoperative donor fascicle NAP amplitude predicts functional recovery following selective ulnar-to-musculocutaneous nerve transfer (Oberlin procedure) for restoration of elbow flexion. **Methods:** This retrospective exploratory observational study included 20 patients who underwent selective ulnar-to-musculocutaneous nerve transfer (Oberlin procedure) with standardized intraoperative neurophysiological mapping and quantitative donor fascicle NAP recording. Functional outcome specific to elbow flexion was assessed at last follow-up using the Medical Research Council (MRC) grading system. Time to first electromyographic evidence of biceps reinnervation was recorded. Associations between intraoperative NAP amplitude and functional, temporal, and clinical variables were analyzed using Spearman’s rank correlation coefficient and non-parametric tests. **Results:** Donor NAP amplitude demonstrated substantial interindividual variability (range 60–400 µV; median 137.5 µV, IQR 87.5–200 µV). No significant associations were observed between NAP amplitude and final MRC grade (ρ = −0.103; *p* = 0.666), time to electromyographic reinnervation (days: ρ = −0.123; *p* = 0.617), patient age, or time from injury to surgery. A moderate negative correlation between NAP amplitude and lesion severity was observed but did not reach statistical significance in this small cohort (ρ = −0.419; *p* = 0.0659). In contrast, shorter time to electromyographic reinnervation was significantly associated with improved final functional outcome (ρ = −0.559; *p* = 0.013). No patient reported postoperative hand weakness. **Conclusions:** In this exploratory cohort, intraoperative donor NAP amplitude was not associated with time to electromyographic reinnervation or final elbow flexion strength following selective ulnar-to-musculocutaneous nerve transfer. Although intraoperative NAP mapping remains essential to confirm axonal continuity and conduction viability of the donor fascicle, NAP amplitude did not demonstrate prognostic value in this cohort and should be interpreted cautiously as an isolated predictor of functional recovery, particularly given the limited sample size and exploratory design. These findings suggest that recovery after selective nerve transfer may be influenced by broader biological determinants, including regenerative timing, rather than by isolated intraoperative amplitude metrics.

## 1. Introduction

Restoration of active elbow flexion remains a central goal in the surgical treatment of brachial plexus injuries. Selective transfer of an ulnar nerve fascicle to the musculocutaneous nerve to reinnervate the biceps brachii, originally described by Oberlin [1], has evolved into a well-established reconstructive procedure, consistently achieving meaningful functional recovery while incurring minimal donor-site morbidity.

Notwithstanding its technical reliability and favorable overall outcomes, predicting the degree and time course of postoperative functional recovery at the individual level remains difficult. Intraoperative neurophysiological monitoring, introduced more than four decades ago [2], provides real-time assessment of nerve conduction across segments implicated in reconstruction. Specifically, intraoperative recording of the nerve action potential (NAP) and compound muscle action potential (CMAP) yields objective electrophysiological data regarding axonal continuity and conduction integrity within the examined nerve segment.

The intraoperative NAP has long been used to confirm axonal continuity in lesions in continuity [3,4]. Its presence has been correlated with preserved neural architecture and a greater likelihood of functional recovery in selected contexts [3,4,5,6]. Beyond confirming continuity, NAP assessment informs intraoperative decision-making in peripheral nerve surgery, guiding identification of viable donor fascicles, defining the extent of non-conductive segments, and supporting decisions regarding reconstruction [4,6,7,8,9,10].

Despite its established role as a marker of axonal continuity, the specific prognostic significance of intraoperative NAP parameters in selective nerve transfer procedures, particularly the Oberlin transfer, remains incompletely defined. Recent evidence demonstrating an association between donor nerve NAP amplitude and final muscle strength after radial-to-axillary nerve transfer [11] suggests that quantitative NAP measures may convey prognostic information extending beyond structural confirmation alone.

Numerous studies have evaluated potential prognostic factors for nerve transfer surgery in the preoperative setting. In contrast, relatively few investigations have focused on intraoperative predictors of functional recovery. Existing intraoperative approaches have primarily emphasized strategies aimed at reducing the regeneration distance to the target muscle, confirming neuronal viability, and optimizing donor fascicle selection during surgery [11,12,13]. Other studies have explored electrophysiological monitoring to estimate the functional capacity of donor nerves, such as in contralateral C7 nerve transfer [14], predictors of postoperative strength in tetraplegia reconstruction [15], or histomorphometric characteristics of donor nerves as potential indicators of functional outcome [16]. Despite these efforts, robust intraoperative markers capable of reliably predicting functional recovery after selective nerve transfer remain incompletely defined.

Importantly, the biological interpretation of intraoperative electrophysiological parameters in selective nerve transfer remains complex. The donor fascicle used in the Oberlin procedure is typically mixed, containing both motor and sensory fibers, and therefore electrophysiological measures such as NAP amplitude may not directly reflect the effective motor axonal contribution transferred to the target muscle. This limitation complicates the interpretation of quantitative NAP parameters as potential prognostic markers.

Consequently, whether intraoperative donor fascicle NAP characteristics are associated with postoperative functional outcomes after ulnar-to-musculocutaneous nerve transfer remains uncertain and represents a clinically relevant knowledge gap.

The present study aimed to evaluate the prognostic value of intraoperative donor nerve NAP amplitude in the recovery of elbow flexion following an Oberlin-type transfer, with particular emphasis on its relationship to final elbow flexion strength and the temporal profile of muscle reinnervation.

## 2. Materials and Methods

### 2.1. Study Design

We performed a retrospective exploratory observational study based on a clinical database of patients who underwent selective nerve transfer to the motor branch of the biceps brachii (Oberlin procedure) to restore elbow flexion following traumatic brachial plexus injury. All patients were treated at two tertiary referral centers between November 2006 and November 2025.

All procedures were performed by the same senior peripheral nerve surgeon, and intraoperative neurophysiological mapping was systematically conducted in every case by the same experienced clinical neurophysiologist using a standardized protocol. Although patients were treated across two centers, the primary surgeon and neurophysiologist remained the same throughout the study period, ensuring procedural and electrophysiological consistency.

The study protocol was approved by the institutional ethics committees of both participating centers (PR250/18 16-May-18; CMT-2018-UCM01 24-May-18). The study was designed, conducted, and reported in accordance with the Strengthening the Reporting of Observational Studies in Epidemiology (STROBE) statement.

### 2.2. Study Population and Construction of the Analytical Cohort

All patients with traumatic brachial plexus injury who underwent nerve transfer for biceps reinnervation with intraoperative neurophysiological mapping during the study period were identified from the institutional databases of the two participating centers. A total of 50 patients met these initial screening criteria. The analytical cohort was then defined according to the predefined eligibility criteria described below. The patient selection process is summarized in the STROBE flow diagram (Figure S1).

**Inclusion criteria:**

- Undergoing selective nerve transfer to the biceps brachii following the classical Oberlin technique.
- Availability of a valid nerve action potential (NAP) recording from the donor ulnar nerve fascicle obtained through intraoperative neurophysiological mapping.
- Fascicular localization and selection performed using compound muscle action potential (CMAP) recording of the ulnar nerve.
- Documented postoperative functional assessment.

**Exclusion criteria:**

Records with incomplete clinical data were excluded. Additional exclusion criteria comprised previous brachial plexus injury, prior nerve transfer for biceps muscle reinnervation, non-traumatic etiology, terminolateral or supercharge nerve transfers, non-interpretable intraoperative electrophysiological recordings, clinical follow-up of ≤6 months, or inability to establish a documented linkage between intraoperative neurophysiological recordings and the corresponding postoperative clinical outcomes in the medical record.

After application of these criteria, the final analytical cohort consisted of 20 patients with available intraoperative NAP recordings and documented postoperative functional evaluation. For analyses involving time to reinnervation, only cases with postoperative electromyographic documentation were included. One patient lacked postoperative EMG data and was therefore excluded from analyses involving time to electromyographic reinnervation.

Functional outcomes were assessed at the last available follow-up visit after a minimum follow-up of 6 months.

Concomitant nerve transfers addressing other functional targets were performed when clinically indicated as part of the reconstructive strategy for brachial plexus injury. Because the objective of the present study was to evaluate the association between donor fascicle NAP amplitude and biceps reinnervation following the Oberlin transfer, analyses were restricted to outcomes directly related to biceps function.

### 2.3. Intraoperative Neurophysiological Mapping

Following surgical exposure for ulnar-to-musculocutaneous nerve transfer (Oberlin procedure), the pneumatic tourniquet was released at least 20 min before electrophysiological assessment. Intraoperative mapping was performed using a 16-channel ISIS IOM system (INOMED^®^, Emmendingen, Germany). All recordings were obtained by the same experienced clinical neurophysiologist using identical monitoring equipment and standardized acquisition parameters throughout the study period.

In all cases, the protocol included recording of the musculocutaneous NAP, CMAPs from the first dorsal interosseous (FDI), abductor digiti minimi (ADM), and flexor carpi ulnaris (FCU) muscles, as well as quantitative assessment of the NAP amplitude of the donor fascicle under standardized conditions. CMAP responses were used to assist fascicular localization within the ulnar nerve. In particular, intraoperative CMAP recording from the FCU muscle was used to confirm functional motor activation of the selected donor fascicle and to support identification of fascicles contributing to elbow flexion reconstruction.

After localization and selection of the donor fascicle (predominantly innervating the FCU) based on CMAP responses, neurolysis was performed to isolate it. NAP recording was obtained using insulated handheld bipolar or tripolar hook electrodes (90°/180°), with an interelectrode distance of 3–5 mm for small fascicles and 5–7 mm for larger structures. Tripolar probes were used to enhance selectivity and minimize stimulation artifacts. Square-wave pulses (0.05–0.2 ms duration, cathodal polarity) were delivered at a stimulation frequency of 1 Hz. Stimulation intensity was progressively increased from low levels until a stable maximal NAP amplitude was obtained. Typical stimulus intensities ranged from 0.1 to 25 mA, although intensities up to 50 mA were occasionally required in cases of severe nerve injury.

NAP recordings were obtained using a bipolar hook electrode positioned at least 3 cm from the stimulation site, with a 12 mm/27 G monopolar subdermal needle electrode serving as ground. Acquisition parameters included a 30 ms sweep (6 ms/division), sensitivity of 30 µV–5 mV/division, and band-pass filtering between 5–10 Hz and 2000–3000 Hz. For each donor fascicle, stimulation was performed repeatedly to obtain several NAP recordings under identical acquisition settings. Multiple responses were acquired, and the amplitude used for analysis corresponded to the largest reproducible and clearly interpretable peak-to-peak NAP response. Recordings contaminated by stimulation artifacts or movement-related noise were discarded, and electrode positioning and grounding were verified before repeat acquisition.

**Figure 1:**
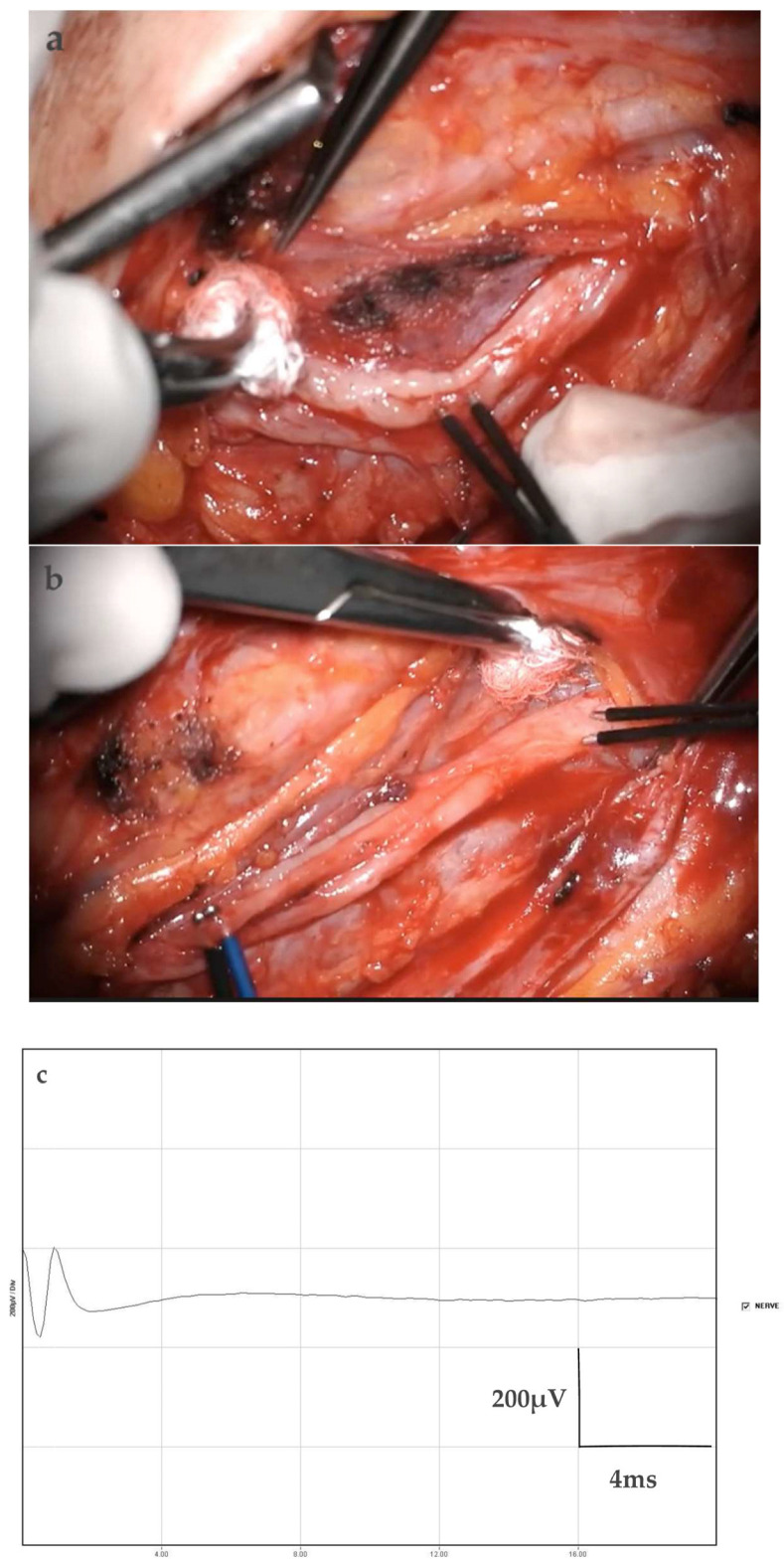
Intraoperative measurement of donor fascicle NAP. (**a**) Selection of the donor fascicle with predominant innervation to the FCU based on intraoperative CMAP recording; (**b**) intraoperative measurement of the donor fascicle NAP; (**c**) representative quantitative intraoperative NAP recording of the donor fascicle. Abbreviations: NAP, nerve action potential; CMAP, compound muscle action potential; FCU, flexor carpi ulnaris.

NAP amplitude was measured peak-to-peak and expressed in microvolts (µV) as a continuous variable (Figure 1). In addition, intraoperative CMAP amplitude of the FCU muscle was recorded and expressed in microvolts (µV).

### 2.4. Postoperative Evidence of Reinnervation

During postoperative follow-up, the time to first electromyographic evidence of reinnervation of the biceps brachii was recorded, expressed in both days and months from the date of surgery. Postoperative electromyographic follow-up was performed according to the institutional clinical protocol, with evaluations generally scheduled at approximately 3 month intervals after surgery. However, the exact timing of EMG assessments varied slightly between patients depending on clinical scheduling and follow-up availability.

Time to first electromyographic evidence of reinnervation was defined as the interval between the date of surgery and the first EMG examination demonstrating motor unit potentials in the biceps brachii muscle.

### 2.5. Functional Outcomes

Elbow flexion was assessed at the last available clinical follow-up using the British Medical Research Council (MRC) grading system, a widely used clinical scale for evaluating motor recovery after peripheral nerve reconstruction. Functional outcomes were recorded after a minimum follow-up of 6 months.

For statistical analyses, the MRC grade was treated as an ordinal variable. For descriptive comparisons, outcomes were additionally dichotomized as nonfunctional (MRC < 3) or functional (MRC ≥ 3), a threshold commonly used to define clinically meaningful elbow flexion. Functional recovery corresponded to the ability to achieve active movement against gravity.

Potential clinical confounders were also recorded, including patient age, level of injury, and time from injury to surgical intervention (expressed in days), and duration of clinical follow-up.

### 2.6. Statistical Analysis

Continuous variables are presented as median and range (minimum–maximum). Distribution normality was assessed using the Shapiro–Wilk test. Given the small sample size and the non-normal distribution of several variables, non-parametric methods were used for all analyses. Spearman’s rank correlation coefficient was used to assess the association between intraoperative NAP amplitude and functional, temporal, and clinical variables; furthermore, 95% confidence intervals were estimated using a bootstrap procedure to improve the robustness of interval estimation in the context of the limited sample size.

Differences in NAP amplitude between functional outcome groups were analyzed using the Mann–Whitney U test (U), and effect size (r) was calculated. Given the exploratory nature of the study and the limited sample size, analyses were restricted to bivariate associations and no multivariable modeling was performed due to the risk of overfitting and unstable parameter estimates in a small cohort. Potential clinical variables, including age, injury level, and time from injury to surgery, were therefore explored individually through correlation analyses rather than incorporated into multivariable models.

No formal correction for multiple comparisons was applied because analyses were considered exploratory and hypothesis-generating; therefore, *p*-values should be interpreted cautiously. Accordingly, non-significant findings should be interpreted as absence of detectable association within this dataset rather than definitive evidence of absence of effect. Statistical analyses were performed using The jamovi project (2026). *jamovi* (Version 2.7) [Computer Software] A two-sided *p* value < 0.05 was considered statistically significant.

## 3. Results

The final analytical cohort comprised 20 patients with valid intraoperative NAP recordings and documented postoperative functional assessment. Follow-up duration demonstrated substantial variability, with a mean of 808.3 ± 693.3 days and a median of 522 days (interquartile range [IQR] 412.5–819.5), corresponding to approximately 26.6 ± 22.8 months (Table S1). In all cases, the etiology of injury was traffic accident. Demographic characteristics are summarized in Table S1.

The intraoperative NAP of the recipient musculocutaneous nerve was 0 µV in all patients. Intraoperative NAP amplitude demonstrated substantial interindividual variability, ranging from 60 to 400 µV, with a median of 137.5 µV (IQR 87.5–200 µV).

Six patients (30%) had a poor functional outcome (MRC < 3), whereas 14 (70%) achieved functional recovery (MRC ≥ 3).

Time to first electromyographic evidence of reinnervation was available for 19 patients. The median time was 149 days (range, 71–268), corresponding to 4 months (range, 2–8).

Age at first visit showed a mean of 37.0 ± 15.6 years and a median of 37.5 years (IQR 26.5–46.3). Time from injury to surgery demonstrated a mean of 216.2 ± 83.4 days with a median of 211 days (IQR 147.5–284.8).

Correlations between intraoperative donor fascicle NAP amplitude and temporal variables, clinical characteristics, and functional outcomes are summarized in Table 1.

Intraoperative NAP amplitude showed no statistically significant correlation with time to electromyographic reinnervation, either when expressed in days (ρ = −0.123; *p* = 0.617; 95% CI: −0.507 to 0.315; n = 19) or in months (ρ = −0.185; *p* = 0.448; 95% CI: −0.595 to 0.260; n = 19).

Likewise, no significant correlations were observed between intraoperative NAP amplitude and patient age (ρ = 0.276; *p* = 0.239; 95% CI: −0.171 to 0.635; n = 20), surgical delay (time from injury to surgery) (ρ = 0.171; *p* = 0.471; 95% CI: −0.345 to 0.661; n = 20), or final elbow flexion strength measured using the MRC scale (ρ = −0.103; *p* = 0.666; 95% CI: −0.529 to 0.408; n = 20).

Comparison of intraoperative NAP amplitude between patients with poor functional outcome (MRC grade < 3) and those with functional recovery (MRC grade ≥ 3) revealed no statistically significant difference (U = 43.0; *p* = 0.967; r = 0.019).

A moderate negative correlation was observed between intraoperative NAP amplitude and level of injury (ρ = −0.419; *p* = 0.0659; n = 20), suggesting a tendency toward lower NAP amplitudes in more extensive brachial plexus lesions; however, this association did not reach conventional statistical significance.

No statistically significant association was identified between intraoperative NAP amplitude and FCU CMAP amplitude (ρ = 0.155; *p* = 0.581; 95% CI: −0.453 to 0.708; n = 15).

Regarding potential confounders, no statistically significant correlation was found between patient age and time to electromyographic reinnervation expressed in days (ρ = 0.363; *p* = 0.127; 95% CI: −0.139 to 0.741; n = 19). Similarly, no significant association was observed between surgical delay and final functional outcome (MRC grade) (ρ = −0.186; *p* = 0.433; 95% CI: −0.573 to 0.274; n = 20). The relationship between surgical delay and time to reinnervation in days showed a weak negative correlation that did not reach statistical significance (ρ = −0.181; *p* = 0.459; 95% CI: −0.585 to 0.263; n = 19) (Table S2).

The relationship between neuropathic pain and functional recovery was also explored. A moderate negative correlation was observed between the presence of neuropathic pain and final MRC grade (ρ = −0.33; *p* = 0.151), although this did not reach statistical significance. In comparative analyses, patients with neuropathic pain demonstrated a higher mean MRC grade (5.0) compared to those without neuropathic pain (3.37); however, this difference was not statistically significant (Mann–Whitney U test, *p* = 0.174). Given the coding of the variable (1 = presence of pain, 2 = absence of pain), the negative correlation indicates that higher values (absence of pain) were associated with lower muscle strength, consistent with the observed group means. These findings should be interpreted cautiously given the limited sample size.

Follow-up duration showed considerable interindividual variability but was not significantly associated with final functional outcome.

No patient reported weakness in hand function.

## 4. Discussion

The present study does not question the clinical utility of intraoperative NAP recording. Rather, it seeks to refine its interpretative framework within the specific context of selective ulnar-to-musculocutaneous nerve transfer for biceps reinnervation, as described by Oberlin (1). In particular, we examined the assumption that greater intraoperative donor NAP amplitude translates into improved postoperative functional recovery [3,6,10,11,17].

Our findings confirm that intraoperative NAP assessment remains essential for verifying axonal continuity and conduction viability of the donor fascicle, thereby ensuring the technical safety of the transfer [7]. However, donor NAP amplitude was not associated with final elbow flexion strength or time to electromyographic evidence of muscle reinnervation. These findings suggest that, in the setting of selective nerve transfer, quantitative conduction amplitude alone may not reliably reflect the effective motor axonal load delivered to the target muscle.

In lesions in continuity, preserved intraoperative NAP has well-established prognostic value because it reflects maintained conduction across a partially injured nerve segment and supports neurolysis over reconstruction [3,7,8,18]. Selective nerve transfer, however, represents a biologically distinct paradigm. In this scenario, postoperative recovery depends not only on preserved conduction within the donor fascicle but also on the number, proportion, and regenerative competence of motor axons successfully redirected to a denervated recipient muscle. Thus, while NAP confirms conduction viability, it does not directly quantify the functional motor substrate transferred.

This distinction is particularly relevant when interpreting amplitude metrics in selective nerve transfer.

Selective nerve transfer should be understood not as restoration of native conduction pathways but as a strategic redistribution of available motor axonal capital toward a denervated target muscle. Within this framework, intraoperative NAP amplitude primarily reflects the presence and synchrony of conducting fibers within the donor fascicle at the time of surgery; however, it does not quantify the effective motor axonal fraction that will ultimately regenerate, reinnervate motor endplates, and integrate functionally within the recipient muscle. Electrophysiological viability may therefore be regarded as a necessary condition for successful transfer; however, it is not sufficient to predict the biological efficiency of motor reinnervation. Functional recovery after nerve transfer is inherently multidimensional, depending on axonal regeneration dynamics, target muscle receptivity, motor unit remodeling, and central adaptive plasticity. Consequently, reliance on isolated amplitude metrics may oversimplify processes governed by complex peripheral and central biological determinants. These mechanistic considerations remain conceptual and were not directly measured in the present study.

Although NAP amplitude is commonly interpreted as reflecting the number and caliber of conducting fibers [19], several neurophysiological mechanisms may attenuate its correlation with functional motor outcomes. Temporal dispersion and partial desynchronization of regenerating axons may reduce amplitude independently of absolute axonal counts. Remyelination status influences conduction velocity and waveform morphology without necessarily altering motor unit potential characteristics. In mixed fascicles, the relative proportion of sensory and motor fibers cannot be inferred from amplitude alone. Collectively, these factors introduce biological variability that may limit the predictive value of amplitude measurements when considered in isolation.

Importantly, this conceptual reframing helps explain why conduction amplitude and final strength recovery may diverge despite technically successful transfer. Accordingly, quantitative NAP amplitude may be more appropriately interpreted as an index of conduction synchrony rather than as a direct proxy for functional motor axon transfer.

Anatomically, the donor fascicle in our cohort was mixed, with predominant FCU innervation. Given the intraneural intermingling of motor and sensory fibers [20,21], selective isolation of purely motor axons is not feasible. Consequently, a robust NAP amplitude may reflect conduction across a mixed fiber population without guaranteeing that a sufficient motor axonal fraction has been transferred to meet the biomechanical demands of elbow flexion. The functional requirements of the biceps brachii, particularly in overcoming gravitational and resistive loads, may exceed the effective motor axonal contribution redirected from a single FCU-dominant fascicle.

Cadaveric axon count studies suggest that a donor-to-recipient motor axonal ratio of approximately 0.7:1 may be sufficient for successful transfer [22]. However, such anatomical estimates do not account for interindividual variability in axonal density, prior axonal degeneration within the recipient nerve, or differences in regenerative efficiency. In our cohort, recipient musculocutaneous NAP was absent in all cases, precluding estimation of baseline axonal substrate. Even when theoretical axon ratios are met, the functional outcome likely depends on dynamic biological processes rather than static intraoperative amplitude metrics.

A moderate negative correlation between donor NAP amplitude and lesion severity approached statistical significance. Although not definitive, this trend may reflect global neurophysiological effects of more extensive plexus injuries. However, the absence of association between amplitude and both reinnervation timing and final MRC grade indicates that lesion extent does not appear to translate linearly into donor conduction amplitude as a functional predictor.

The absence of a statistically detectable association between donor NAP amplitude and functional recovery should also be interpreted in the context of biological variability and methodological constraints.

Functional recovery after nerve transfer is influenced by multiple interacting biological determinants. In addition to the timing of reinnervation, factors such as duration of denervation and motor endplate preservation, the biological condition of the recipient muscle, donor nerve axonal load and regenerative capacity, patient age, and systemic factors may all contribute to the final outcome. Although nerve transfer techniques are designed to reduce regeneration distance compared with proximal nerve reconstruction, variability in the effective regeneration pathway and in muscle receptivity may still influence recovery.

Experimental and clinical evidence suggests that motor endplate degeneration progresses with prolonged denervation, with increasingly limited reversibility beyond several months. In this context, a single intraoperative neurophysiological measurement is unlikely to fully capture the complex biological processes that ultimately determine functional recovery.

Although earlier surgical intervention is generally associated with improved outcomes, the absence of a significant association between surgical delay and functional outcome in our cohort should be interpreted cautiously, particularly given the limited sample size and variability in clinical presentation.

The strongest association observed was the inverse correlation between time to electromyographic reinnervation and final MRC grade (ρ = −0.559; *p* = 0.013). This finding reinforces the biological principle that timely muscle reinnervation remains a primary determinant of functional recovery [6,23]. Rather than implying hierarchical superiority over conduction amplitude, these results suggest that regenerative timing may exert a clinically meaningful influence on recovery, independent of baseline intraoperative amplitude metrics. Muscle receptivity to reinnervation declines with prolonged denervation due to motor endplate degeneration, fibrosis, and reduced satellite cell responsiveness [19,24]. Thus, regenerative timing appears to exert substantial influence on functional outcomes.

A non-significant trend toward higher MRC grades was observed in patients with neuropathic pain. Although this finding should be interpreted cautiously given the limited sample size and potential confounding, it may suggest that neuropathic pain does not necessarily reflect worse functional recovery and could be associated with partially preserved or regenerating neural pathways.

Functional recovery after nerve transfer also requires central nervous system adaptation. Adaptive cortical reorganization and motor relearning following cross-innervation have been documented [25,26]. Such central plasticity introduces additional variability that cannot be captured by peripheral conduction amplitude alone, further explaining the dissociation between intraoperative electrophysiological parameters and ultimate strength recovery.

Importantly, demonstrating the absence of a statistically detectable prognostic association is clinically relevant. Overinterpretation of intraoperative NAP amplitude could theoretically lead to unwarranted preference for higher-amplitude fascicles without clear functional benefit. Our findings support a cautious interpretation: the presence of a viable NAP confirms technical suitability of the donor fascicle, but its amplitude should not independently guide prognostic expectations regarding elbow flexion strength.

Our study has several limitations. First, its retrospective observational design limits causal inference and relies on the completeness of clinical documentation; postoperative EMG data were unavailable for one patient, resulting in a slightly reduced sample size for the reinnervation analysis. Second, the small sample size restricts statistical power, particularly for detecting small-to-moderate associations; therefore, non-significant findings should be interpreted as lack of detectable association within this exploratory cohort rather than proof of biological absence. Third, the study period spans nearly two decades, and unmeasured temporal heterogeneity in rehabilitation practices, follow-up strategies, or subtle acquisition differences may exist. However, surgical procedures and intraoperative neurophysiological recordings were performed using a standardized protocol by the same surgeon and neurophysiologist across the two participating centers, both with more than 10 years of prior experience at the beginning of the study period, which likely reduced technical variability. Fourth, concomitant nerve transfers addressing additional functional deficits were performed in a substantial proportion of patients as part of the reconstructive strategy for brachial plexus injury and may represent a potential source of confounding when interpreting functional recovery. In addition, variability in the anatomical extent of brachial plexus injury may also contribute to heterogeneity in functional recovery. However, the present analyses focused specifically on outcomes related to biceps reinnervation following the Oberlin transfer, and multivariable modeling was not statistically appropriate due to the risk of overfitting in this small cohort. Fifth, postoperative EMG timing was not fully standardized (planned approximately 3-month intervals after surgery but variable in practice), and the recorded time to first electromyographic reinnervation may therefore reflect both biological regeneration and variability in assessment intervals. Sixth, outcome assessment relied on the ordinal MRC grading system, which may be insufficiently sensitive to detect subtle between-patient differences, and dichotomization may reduce the amount of available information. Finally, donor-site morbidity was not systematically quantified using objective measures such as dynamometric strength testing, including grip and pinch strength assessment, or standardized sensory assessments (e.g., two-point discrimination) in all patients. Despite these limitations, the present study provides a standardized intraoperative electrophysiological dataset and exploratory evidence regarding the potential prognostic relevance of donor fascicle NAP amplitude in selective nerve transfer.

Future prospective studies incorporating quantitative dynamometry, extended follow-up, and multivariable modeling integrating electrophysiological, temporal, and clinical variables may better define the multidimensional determinants of functional recovery after selective nerve transfer.

## 5. Conclusions

Intraoperative NAP mapping remains essential for confirming axonal continuity and conduction viability of the donor fascicle during selective nerve transfer. In this exploratory cohort, donor NAP amplitude was not associated with time to electromyographic reinnervation or final elbow flexion strength following selective ulnar-to-musculocutaneous nerve transfer. NAP amplitude did not demonstrate prognostic value in this cohort and should therefore be interpreted cautiously as an isolated predictor of functional recovery, particularly given the limited sample size. These findings suggest that recovery after selective nerve transfer may be influenced by broader biological determinants, including regenerative timing, rather than by isolated intraoperative amplitude metrics. Our findings support the use of intraoperative NAP primarily as a tool for confirming donor fascicle viability rather than for prognostic stratification.

## Figures and Tables

**Table 1:** Correlations between intraoperative donor fascicle NAP amplitude and temporal variables, clinical characteristics, and functional outcomes (Spearman’s Rank Correlation Coefficient).

Variable	ρ	*p*	95% CI
Final MRC	−0.103	0.666	−0.529 to 0.408
Time to Reinnervation (days)	−0.123	0.617	−0.507 to 0.315
Time to Reinnervation (months)	−0.185	0.448	−0.595 to 0.260
Age	0.276	0.239	−0.171 to 0.635
Surgical delay (days)	0.171	0.471	−0.345 to 0.661
FCU CMAP amplitude	0.155	0.581	−0.453 to 0.708
Level of brachial plexus injury	−0.419	0.0659	−0.757 to 0.097

Note: n = 20 unless otherwise indicated. Analyses involving time to electromyographic reinnervation were performed in 19 patients because postoperative EMG data were unavailable in one case. Correlations including FCU CMAP amplitude were available in 15 cases due to incomplete intraoperative CMAP recordings. Abbreviations: NAP, nerve action potential; CMAP, compound muscle action potential; FCU, flexor carpi ulnaris; MRC, British Medical Research Council muscle strength grading system.

## Data Availability

The datasets generated and/or analyzed during the current study are not publicly available due to patient privacy and ethical restrictions but are available from the corresponding author on reasonable request and subject to Institutional Review Board approval.

## Supplementary Materials

The following supporting information can be downloaded at: https://www.mdpi.com/article/10.3390/jcm15072521/s1, Figure S1. Flow diagram of patient selection and inclusion in the study cohort; Table S1: Baseline demographic and clinical characteristics of the analytical cohort; Table S2: Spearman correlation analyses between clinical variables.

## Abbreviations

The following abbreviations are used in this manuscript:

CMAP	Compound muscle action potential
NAP	Nerve action potential
FCU	Flexor carpi ulnaris
FDI	First dorsal interosseous
ADM	Abductor digiti minimi
MRC	British Medical Research Council muscle strength grading system
