# Supplementary material for: Intraoperative Nerve Action Potential Amplitude and Functional Recovery After Selective Ulnar-to-Musculocutaneous Nerve Transfer (Oberlin Technique)
Source: J Clin Med. 2026 Mar 26;15(7):2521. doi: 10.3390/jcm15072521 (PMC13073336; doi:10.3390/jcm15072521)
Supplement: Supplementary file 1 [file jcm-15-02521-s001.zip › Table S1 and S2.pdf]

Table S1. Baseline demographic and clinical characteristics of the analytical cohort
Overall Cohort (n = 20)

Variable	Value
Age at first visit (years), mean ± SD	37.0 ± 15.6
Age at first visit (years), median (IQR)	37.5 (26.5–46.3)
Sex	
- Male, n (%)	19 (95.0%)
- Female, n (%)	1 (5.0%)
Affected side	
- Right, n (%)	12 (60.0%)
- Left, n (%)	8 (40.0%)
Time from injury to surgery	216.2 ± 83.4 days (7.10 ± 2.74 months)
Time from injury to surgery, median (IQR)	211 days (147.5–284.8) [6.93 (4.85–9.36) months]
Follow-up duration	808.3 ± 693.3 days (26.57 ± 22.79 months)
Follow-up duration, median (IQR)	522 days (412.5–819.5) [17.16 (13.56–26.93) months]
Concomitant nerve transfers performed, n (%)	16 (80.0%)
Ulnar territory dysesthesias, n (%)	2 (10.0%)

All injuries were secondary to traffic accidents.

Stratified by level of brachial plexus injury

Injury level	n	Age (years), mean ± SD	Time to surgery (months), mean ± SD	Donor fascicle NAP (μV), mean ± SD
**C5–C6**	4	28.0 ± 9.1	7.9 ± 2.2	206.3 ± 96.6
**C5–C7**	12	41.4 ± 16.6	7.1 ± 2.6	182.5 ± 112.6
**C5–C8**	2	40.5 ± 17.7	4.0 ± 0.1	80.0 ± 28.3
**C5–T1**	2	25.0 ± 11.3	8.5 ± 5.2	90.0 ± 14.1

Abbreviations: SD, standard deviation; NAP, nerve action potential.

Table S2. Spearman correlation analyses between clinical variables

Comparison	n	ϱ	p-value	95% CI
Age and reinnervation time (days)	19	0.363	0.127	−0.139 to 0.741

Time to surgery and MRC score	20	−0.186	0.433	−0.573 to 0.274
Time to surgery and reinnervation time (days)	19	−0.181	0.459	−0.585 to 0.263
Reinnervation time (days) and MRC score	19	−0.559	0.013	−0.844 to −0.114
Neurophatic pain and MRC score	20	-0.333	0.151	−0,628 to −0,271

Abbreviations: MRC, Medical Research Council muscle grading system.
