# Supplementary figures and images for: Intraoperative Nerve Action Potential Amplitude and Functional Recovery After Selective Ulnar-to-Musculocutaneous Nerve Transfer (Oberlin Technique)
Source: J Clin Med. 2026 Mar 26;15(7):2521. doi: 10.3390/jcm15072521 (PMC13073336; doi:10.3390/jcm15072521)

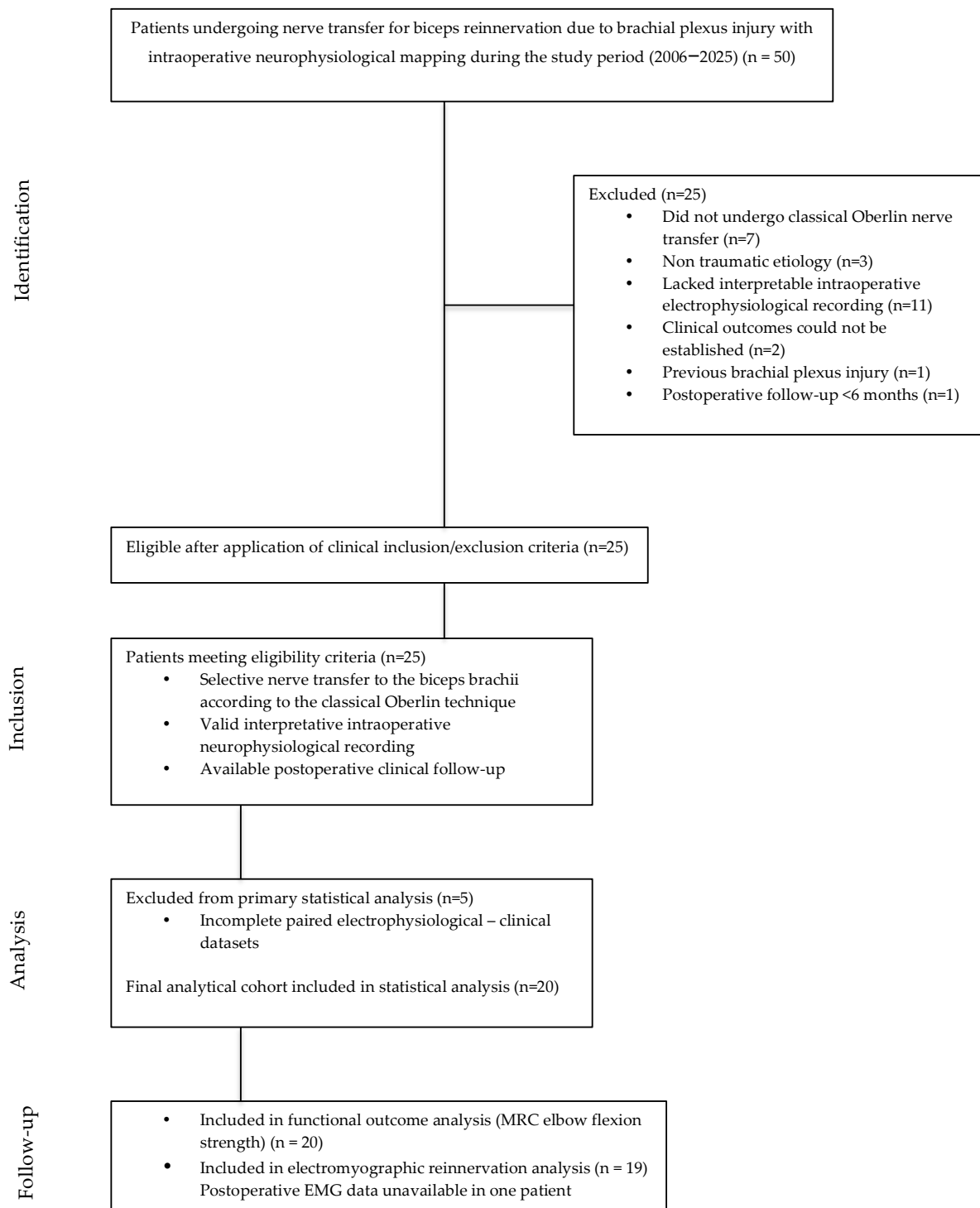

**Figure S1. Flow diagram of patient selection and inclusion in the study cohort**

Supplement: Supplementary file 1 [file jcm-15-02521-s001.zip › Figure S1. Flow diagram of patient selection and inclusion in the study cohort.pdf]
